# Acute vestibular syndrome induced by supratentorial stroke: clinical features and network compensation mechanisms

**DOI:** 10.3389/fneur.2025.1690103

**Published:** 2025-12-02

**Authors:** Songbin Pan, Yisi Zhang, Xian Ding, Xiaofeng Pan, Xiaoguang Chen, Xiaoqiang Lv, Hong You, Junyv Zhu

**Affiliations:** 1 Department of Neurology, Wuhan No.1 Hospital, Wuhan, China; 2 Department of Neurology, Huangshi Central Hospital, Affiliated Hospital of Hubei Polytechnic University, Huangshi, China; 3 School of Medicine, Jianghan University, Wuhan, China

**Keywords:** hemispheric infarction, acute vestibular syndrome, vestibular cortex, neuro-otorhinolaryngology, neuro-ophthalmology

## Abstract

**Introduction:**

It remains unclear which vestibular symptoms and signs are associated with supratentorial stroke presenting as acute vestibular syndrome, and whether there is hemispheric predominance in the manifestation of these symptoms and signs.

**Methods:**

This prospective study aims to further characterize the clinical features and explore the underlying mechanisms of acute vestibular syndrome (AVS) in patients caused by supratentorial stroke by integrating the findings from neuro-otological and neuro-ophthalmological examinations.

**Results:**

This study ultimately included 13 patients with AVS were confirmed supratentorial stroke by diffusion weighted imaging(DWI). Infarction involved only the cortical areas in 7 patients, only the subcortical structures in 5 patients, and both regions in 1 patient. 61.5% (8/13) of patients exhibited at least one abnormal neuro-otological or neuro-ophthalmological finding, including smooth pursuit test (*n* = 2), optokinetic test (*n* = 3), saccades test (*n* = 2), caloric test (*n* = 2), video head impulse tests (*n* = 3).

**Discussion:**

Lesions showed left hemispheric predominance overall, but no clear hemispheric predominance for specific symptoms (50% of vertigo lesions were right-sided). Vertigo was associated with cortical lesions (4/4), while dizziness more often involved subcortical structures (6/9), suggesting different mechanisms: acute disruption of vestibular-visual integration versus impaired vestibulo-cortical signaling via basal ganglia-thalamo-cortical circuits. In summary, AVS induced by supratentorial stroke results from the interplay between multimodal network impairment and dynamic compensation. The study highlights the need to incorporate detailed vestibular testing beyond HINTS to avoid missing supratentorial stroke in AVS patients, despite limitations like small sample size.

## Introduction

Acute vestibular syndrome (AVS) refers to a disorder characterized by a single episode of sudden-onset vestibular symptoms and signs (e.g., vestibular neuritis or acute stroke) (1). Conventional wisdom held that AVS caused by stroke is typically located in the posterior circulation (2, 3).

Previous studies have indicated that several independent temporoparietal networks constitute the vestibular cortex, which encompasses the insular gyrus, intraparietal sulcus, inferior parietal lobule, superior temporal gyrus, middle temporal gyrus, precentral gyrus, middle frontal gyrus, hippocampus, cingulate gyrus, putamen and thalamus, whose core region is located in the parieto-insular vestibular cortex (PIVC) (4–6). Currently, only a limited number of studies have described AVS induced by supratentorial stroke (7–10). Whether there is hemispheric predominance in these vestibular symptoms remains inconclusive and requires further investigation.

We conducted a prospective study aiming to further characterize the clinical features and explore the underlying mechanisms of AVS caused by supratentorial stroke by combining the results of neuro-otological and neuro-ophthalmological examinations.

## Methods

This study was conducted in accordance with the Declaration of Helsinki and was approved by the Ethics Committee of Wuhan No.1 Hospital. Written informed consent was obtained from all participants or their legal guardians.

We prospectively and consecutively enrolled patients with ischemic stroke admitted to the department of neurology of Wuhan No.1 Hospital between June 2021 and July 2024. All enrolled patients presented with AVS, who fulfilled the Barány Society diagnostic criteria of a single episode of sudden onset vestibular symptoms and signs (1). Posterior circulation stroke cases were excluded by diffusion weighted imaging (DWI). Patients with cognitive impairment (Mini-Mental State Examination <24/30) (8) or those declining study participation were excluded.

A detailed medical history was collected upon patient admission, with special attention to acute vestibular symptoms (vertigo, dizziness, vestibulovisual symptoms, and postural symptoms) as defined by the Barány Society Committee in 2009 (1). Subsequently, standardized neuro-ophthalmological and neuro-otological examinations were performed by professional neurologists, including saccade tests, smooth pursuit tests, optokinetic tests, gaze tests, static postural tests, video head impulse tests (vHIT), caloric tests and cervical vestibular evoked myogenic potentials (cVEMP). All examinations were performed by the same specialty-trained neurologist within 24 h of patient admission.

Using the video-nystagmography (VNG) instrument (Shanghai Yougeng ZT-VNG-II) to acquire eye movement states, and employing relevant software to analyze and process the image signals. 1. Pathological spontaneous nystagmus is defined as slow-phase velocity (SPV) > 7°/s. 2. Gaze at upper, lower, left, and right 20° positions for at least 20 s each. If nystagmus with SPV > 7°/s appears, it is defined as pathological gaze-evoked nystagmus. 3. In the saccadic test, normal results are defined as saccadic velocity >75°/s, accuracy between 70 and 110%, and latency <260 ms. Results deviating from these parameters are classified as abnormal, abnormalities include undershoot (accuracy <70%), overshoot (accuracy >110%), slow saccades (saccadic velocity <75°/s) and disorganized saccades (latency >260 ms). 4. In the smooth pursuit test, Type III and IV curves associated with nystagmus are classified as abnormal, while Type I and II curves are classified as normal. 5. The optokinetic test is considered abnormal if any of the following are abnormal: direction of nystagmus, amplitude of nystagmus or symmetry of bidirectional nystagmus. 6. Abnormal static balance posture test results are defined as abnormalities in either the velocity or amplitude dimension. In the velocity dimension, abnormality is indicated by proprioception <85%, vestibular sensation <55% or vision <72%. In the amplitude dimension, abnormality is indicated by proprioception <90%, vestibular sensation <72% or vision <70%. 7. In the vHIT, the patient is positioned seated while fixating on a target at 1.2 meters. Following software-specific calibration procedures, a series of horizontal head impulses are administered in all directions. Concurrently, vertical head impulses are applied in the left anterior-right posterior (LARP) and right anterior-left posterior (RALP) semicircular canal planes. A minimum of 20 valid impulses per semicircular canal must be recorded, with head velocities maintained between 100 and 250°/s. Vestibulo-ocular reflex (VOR) gain is derived from the instantaneous ratio of eye movement angular velocity to head movement angular velocity. Abnormal findings are defined as horizontal semicircular canal gain values < 0.8 or vertical semicircular canal gain values < 0.7 (11–13).

The caloric test involves alternately irrigating both ears with 8 L of cold (24 °C) air and warm (50 °C) air for 60s each while recording nystagmus in both eyes. Vestibular function is calculated using Jongkees’ formula, with an interaural difference reaching or exceeding 25% being defined as Canal Paresis (14).

The cVEMP test was performed using the Eclipse EP25 VEMP Evoked Potential System (Interacoustics A/S, Middelfart, Denmark). Recording electrodes were placed at the midpoint of the sternocleidomastoid muscles, and reference electrodes positioned on the sternoclavicular joint surface. Subjects were placed in a supine position while maintaining approximately 30° head elevation to ensure bilateral sternocleidomastoid muscle tension. A 500 Hz tone burst was delivered as the acoustic stimulus, and the resulting potentials generated by ipsilateral sternocleidomastoid muscle activity were recorded. An interaural asymmetry ratio below 35% was defined as normal (15).

All patients received examination with the MAGNETOM Skyra 3 T Superconducting Magnetic Resonance Imaging System, with a scan slice thickness of 5 mm. The imaging diagnosis of acute ischemic stroke was jointly performed by radiologists and neurologists, defining areas that showed hyperintensity on DWI and hypointensity on apparent diffusion coefficient (ADC) maps as infarct foci.

## Results

**Figure 1:**
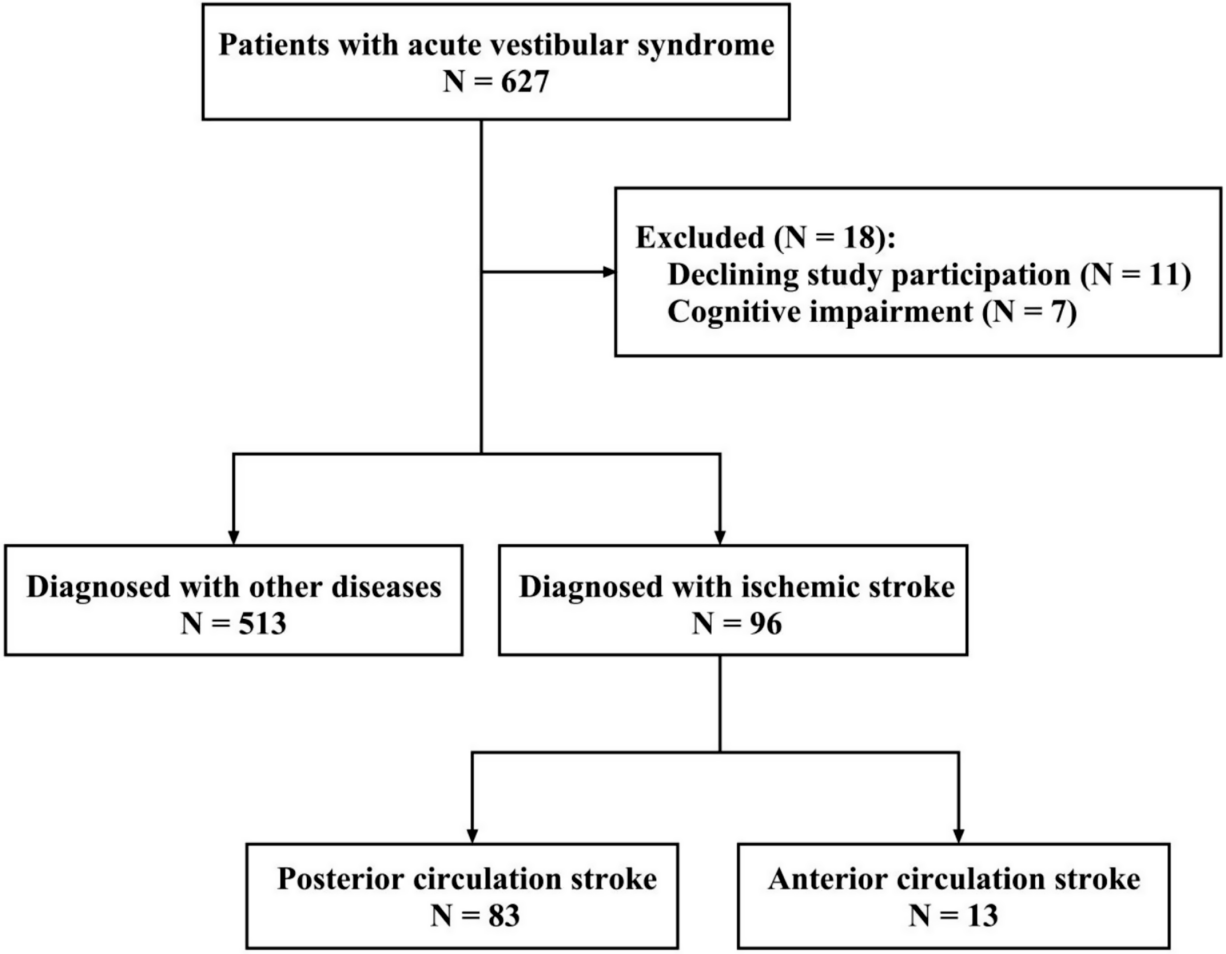
Flowchart of study subject inclusion.

This study ultimately included 13 patients (9 males and 4 females) (Figure 1). These patients ranged in age from 42 to 82 years, and none had peripheral vestibular disease at stroke onset. 4 patients presented with vertigo, and 9 patients presented with dizziness. Among them, 6 patients had significant unsteadiness while walking, and 4 patients had significant nausea or vomiting. None of the patients exhibited other focal neurological deficit signs. Symptoms lasted for several hours (<24 h) in 7 patients, while they lasted for several days (>24 h) in 6 patients. All 13 patients were right-handed aaccording to the assessment of Edinburgh Handedness Inventory (16) (Table 1).

**Table 1:** Characteristics of 13 patients with supratentorial stroke.

Case	Age	Sex	Handedness	Clinical findings	Duration	Lesion side	Anatomical localization of lesions on MRI
1	60	M	R	Dizziness	Hours	R	Superior temporal gyrus
2	79	M	R	Dizziness, unsteadiness on walking	Hours	L	Putamen
3	60	M	R	Vertigo, nausea	Days	R	Middle frontal gyrus
4	60	M	R	Dizziness, unsteadiness on walking	Hours	L	Angular gyrus
5	69	F	R	Vertigo, nausea, vomiting	Days	R	Middle frontal gyrus
6	76	M	R	Dizziness, unsteadiness on walking	Hours	L	Corona radiata
7	42	M	R	Dizziness, nausea, vomiting	Hours	L	Centrum semiovale
8	82	F	R	Vertigo, nausea	Days	L	Precentral gyrus
9	71	M	R	Vertigo, unsteadiness on walking	Hours	L	Angular gyrus
10	76	F	R	Dizziness	Days	L	Angular gyrus
11	62	M	R	Dizziness, unsteadiness on walking	Days	L	Putamen
12	74	M	R	Dizziness, unsteadiness on walking	Hours	L	Adjacent to the posterior horn of the lateral ventricle
13	76	F	R	Dizziness	Days	R	Parahippocampal gyrus, angles of the fornix, posterior limb of the internal capsule

Most patients exhibited involvement of the left cerebral hemisphere (9 patients with left hemisphere involvement and 4 patients with right hemisphere involvement).

**Figure 2:**
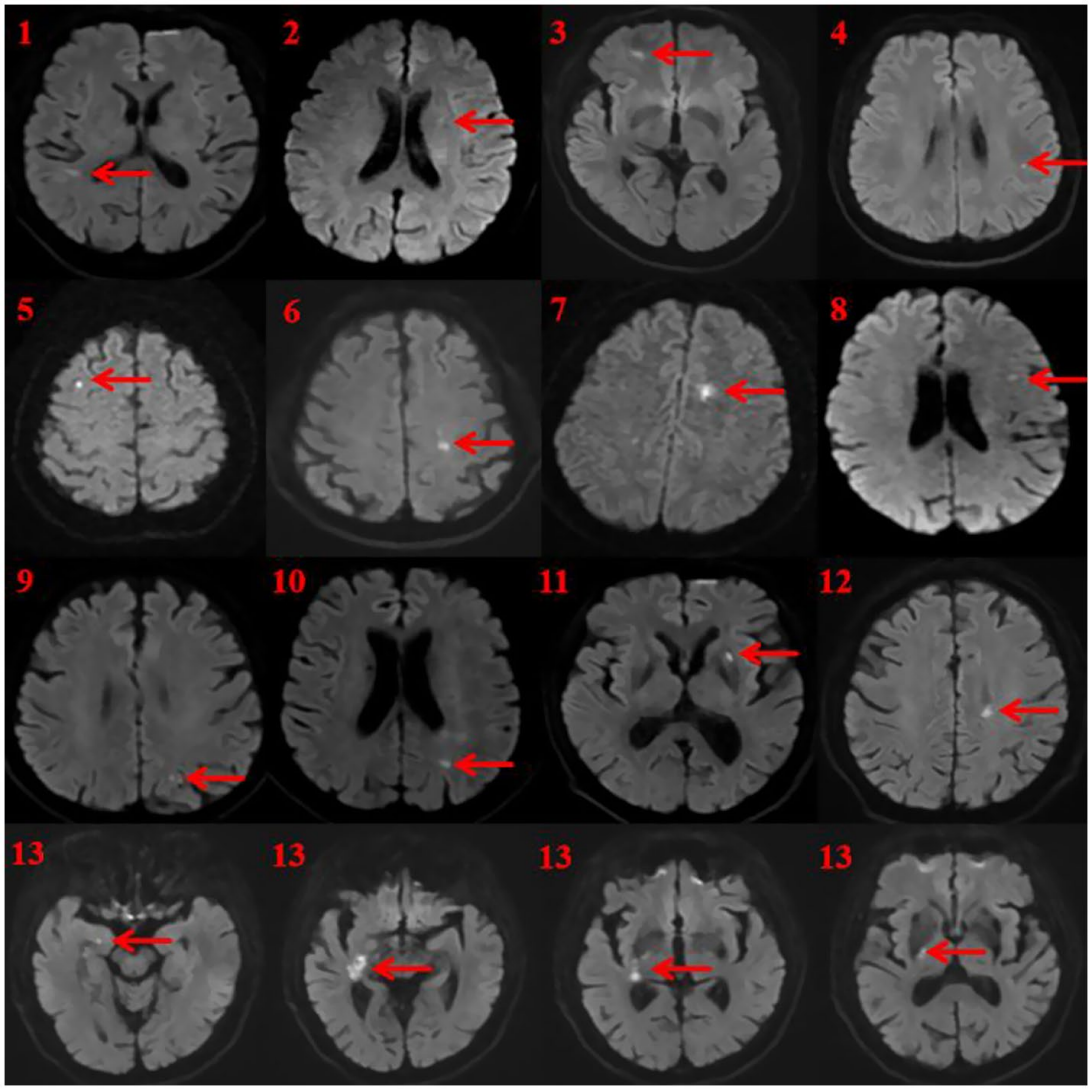
MRI lesions of 13 patients with hemispheric stroke. The infarct is shown at the arrow. The infarctions were located in various brain regions. Specifically, the angular gyrus was involved in three cases (Cases 4, 9, and 10). The middle frontal gyrus (Cases 3 and 5) and the putamen (Cases 2 and 11) were each affected in two cases. The remaining infarctions were found in single locations: the superior temporal gyrus (Case 1), corona radiata (Case 6), centrum semiovale (Case 7), precentral gyrus (Case 8), an area adjacent to the posterior horn of the lateral ventricle (Case 12), and multiple structures including the parahippocampal gyrus in Case 13.

Infarction involved only the cortical areas in 7 patients (cases 1, 3, 4, 5, 8, 9, 10), only the subcortical structures in 5 patients (cases 2, 6, 7, 11, 12), and case 13 had involvement in both areas. The specific locations included angular gyrus (cases 4, 9, 10), middle frontal gyrus (cases 3, 5), superior temporal gyrus (case 1), precentral gyrus (case 8), parahippocampal gyrus (case 13), posterior limb of the internal capsule (case 13), putamen (cases 2, 11), centrum semiovale (cases 6, 7), adjacent to the posterior horn of the lateral ventricle (case 12) (Table 1; Figure 2).

Among the 7 patients in the cortical group, 3 presented with dizziness and 4 with vertigo; symptoms lasted several hours in 3 patients and several days in 4, with lesions located on the left side in 4 and the right side in 3. All 5 patients in the subcortical group had dizziness with left-sided lesions, and while symptoms persisted for several hours in 4 cases, only one patient had symptoms lasting several days.

**Figure 3:**
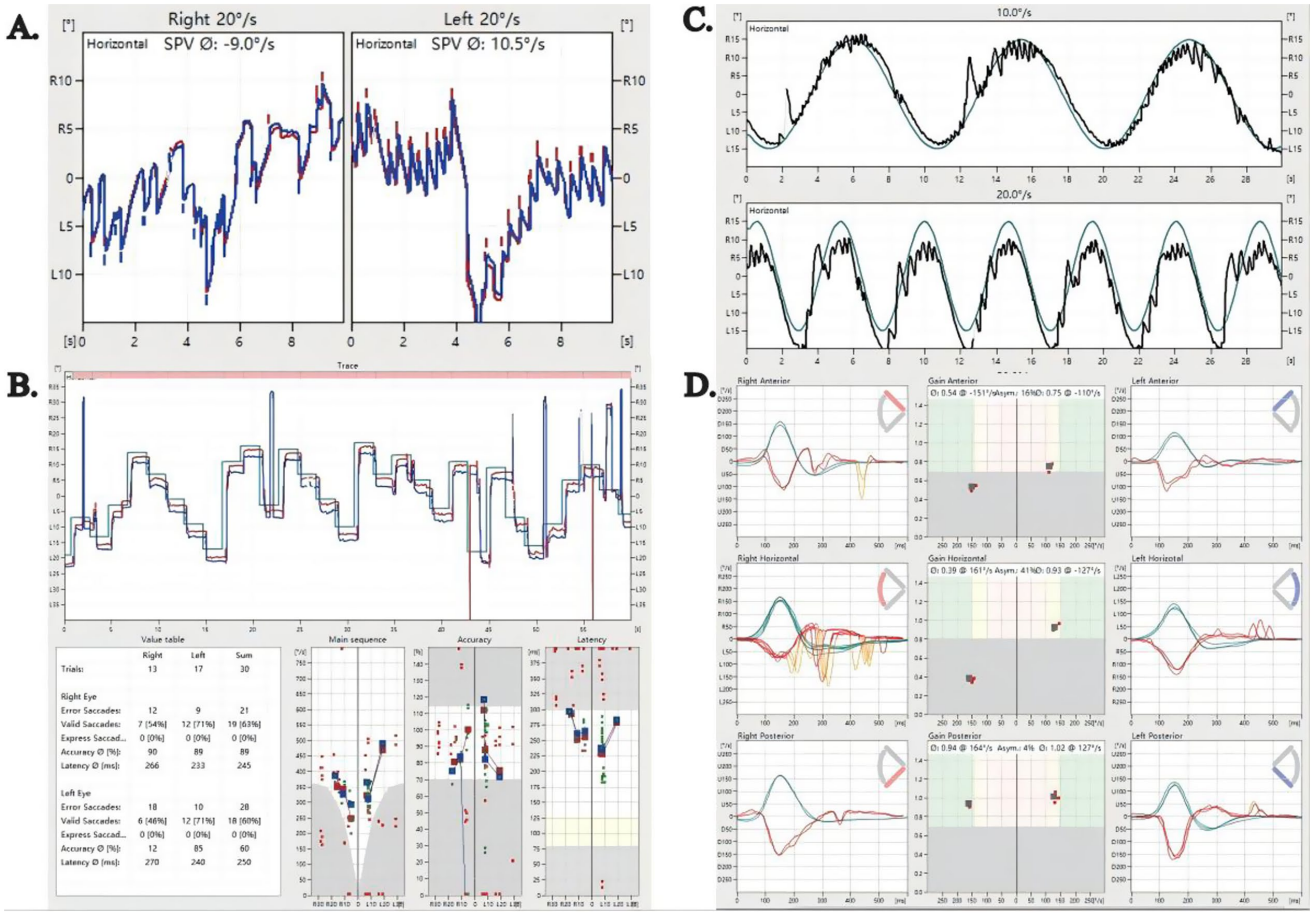
Neuro-ophthalmological and neuro-otological findings in four patients. **(A)** Optokinetic test in Case 4, indicating bilateral reduction in optokinetic nystagmus gain. The stimulus moved at 20°/s in both rightward and leftward directions. The average slow-phase velocity was −9.0°/s (negative sign indicates leftward direction) for rightward movement and 10.5°/s for leftward movement. **(B)** Saccade test in Case 2 showed reduced accuracy bilaterally, with normal saccadic velocity and latency, indicating bilateral undershoot saccades. **(C)** Smooth pursuit test in Case 7, revealing a Type III abnormal curve. The tracking curve, obtained using target velocities of 10.0°/s and 20.0°/s, was non-smooth and stepped, with multiple saccadic waves superimposed on it. **(D)** Video head impulse test in Case 10, demonstrating impaired high-frequency vestibular function on the right side. This primarily affected the horizontal canal (gain: 0.39; normal >0.8) and the anterior canal (gain: 0.54; normal >0.7). The right posterior canal and all left semicircular canals showed normal gain values. Significant asymmetry was observed in the horizontal (41%) and anterior (16%) canals, further supporting right-sided dysfunction.

All 13 patients had normal static postural tests and cVEMP. Abnormalities were found in partial neuro-ophthalmological and neuro-otological examinations in 8 patients, including smooth pursuit test (*n* = 2), optokinetic test (*n* = 3), saccades test (*n* = 2), caloric test (*n* = 2), vHIT (*n* = 3). For brevity, we have omitted the specific values of normal results and only presented abnormal results (Table 2; Figure 3).

**Table 2:** Neuro-ophthalmological and neuro-otological examination results of 13 patients with hemispheric stroke.

Case	Abnormal results of neuro-ophthalmological and neuro-otological tests
1	None
2	Saccade test: bilateral horizontal saccadic undershoot. Accuracy(R 63%, L 60%) Caloric test: bilateral horizontal semicircular canal hypofunction. R50 + R24 = 3°/s, L50 + L24 = 5°/s, CP(R) = 29%
3	None
4	Optokinetic test: bilateral horizontal optokinetic abnormalities. R:−9.0°/s, L:10.5°/s
5	None
6	None
7	Smooth pursuit test: the horizontal direction is type III curve. Optokinetic test: bilateral horizontal optokinetic abnormalities. R:−20.3°/s, L:13.1°/s
8	Video head impulse test: decline in high-frequency function of bilateral horizontal and posterior semicircular canals. A(R 0.74, L 0.86), H(R 0.73, L 0.74), P(R 0.62, L 0.56)
9	Saccade test: bilateral horizontal saccadic undershoot. Accuracy (R 66%, L 61%)
10	Video head impulse test: decline in high-frequency function of the right horizontal and anterior semicircular canals. A(R 0.67, L 0.91), H(R 0.62, L 0.97), P(R 0.94, L 0.93)
11	None
12	Video head impulse test: decline in high-frequency function of the left horizontal semicircular canal. A(R 0.81, L 0.92), H(R 0.98, L 0.74), P(R 0.75, L 0.95) Caloric test: the left horizontal semicircular canal hypofunction. R50 + R24 = 13°/s, L50 + L24 = 0°/s, CP(R) = 100%
13	Smooth pursuit test: the horizontal and vertical direction are both type III curves. Optokinetic test: bilateral horizontal optokinetic abnormalities. R:−16.8°/s, L:11.2°/s

For brevity, only abnormal findings are listed; normal/negative findings have been omitted.

A, anterior semicircular canals; H, horizontal semicircular canal; L, left; P, posterior semicircular canals; R, right.

## Discussion

Among the 13 patients with acute supratentorial stroke in this study, 30.8% (4/13) presented with vertigo, 69.2% (9/13) presented with dizziness, and 46% (6/13) experiencing significant gait instability. Previous studies have found that posterior circulation lesions are more likely to cause autonomic symptoms such as nausea/vomiting (2, 17). However, Park et al. proposed that supratentorial strokes are more likely to provoke spatial disorientation rather than typical vertigo syndromes (7).

In this study, the incidence of nausea/vomiting was relatively low at only 30.8% (4/13), supporting the ‘atypical’ nature of cortical vestibular symptoms. Multiple previous studies suggest the right hemisphere holds dominance in spatial attention and vestibular integration, possibly overlapping with the neural mechanisms underlying vestibular-spatial neglect (e.g., right parietal lesions more frequently cause neglect) (9, 18). Shuichiro Eguchi and colleagues included 930 patients with acute cerebral infarction to investigate whether acute hemispheric stroke causes vestibular symptoms and to analyze the predilection for lesion hemispheres. Through group comparisons, they concluded that right hemisphere lesions are more likely to cause vestibular symptoms (9). However, the culprit lesions showed a distinct left hemispheric predominance in our study. Possible reasons for this opposite phenomenon include small sample bias or population heterogeneity. Conversely, some studies report no clear hemispheric predominance (7, 8). Park et al. conducted a study to determine the anatomical correlates of AVS in supratentorial stroke. They consecutively enrolled 1,301 patients with supratentorial stroke, 48 of whom presented with AVS. The results showed no lateralization of the causative lesions in these patients (7). One possible explanation is that right-hemispheric dominance might apply only to specific symptoms like vertigo, whereas dizziness or imbalance may be modulated by bilateral networks, leading to discrepancies across studies. In this study, 50% of vertigo patients had right-sided lesions, while only 22% of dizziness patients had right-sided lesions. Regrettably, this study only included a sample size of 13 cases, which limited the statistical power and made it difficult to draw rigorous conclusions, only allowing for speculation that more strongly supports the conclusion of non-hemisphericization.

Vestibular information processing relies on a cortico-subcortical network, including the PIVC, superior temporal gyrus, basal ganglia, thalamus and so on (19). The PIVC is considered the core region for vestibular processing, integrating multimodal signals from the vestibular nuclei, cerebellum, visual system and somatosensory system (4, 19). Stroke lesions affecting these regions may cause vertigo, balance disorders or spatial disorientation. In supratentorial stroke patients with vestibular symptoms, Eguchi et al. found lesions frequently involved the insula, parietal operculum, superior temporal gyrus and basal ganglia (9). Concurrently, Park et al. reported that 50% of causative lesions were located in the basal ganglia/thalamus/internal capsule, while only 10% involved the PIVC (7). In contrast, isolated PIVC lesions are often asymptomatic (20). Following acute injury, the unaffected hemisphere or adjacent regions may compensate for vestibular signal processing through functional reorganization, resulting in transient or mild symptoms (21). This compensatory mechanism may explain why isolated PIVC lesions rarely cause vertigo. These findings suggest the vestibular network is extensive and symptoms manifest only when multiple regions are concurrently damaged. In this study, 46% (6/13) of causative lesions were located in subcortical regions (putamen, posterior limb of internal capsule, semioval center), indicating that dysfunction in the basal ganglia-thalamo-cortical circuit may indirectly induce symptoms by affecting spatial orientation networks—consistent with Dieterich’s proposed “distributed compensation failure” mechanism (18). All lesions in vertigo patients were located in the cortex (4/4), while two-thirds (6/9) of dizziness patients had lesions involving subcortical structures. Regarding the mechanism underlying the heterogeneity of vertigo and dizziness symptoms, we speculate that vertigo is often related to an acute lesion in the temporo-parietal junction causing abrupt disruption of vestibular-visual integration, and lesions in the basal ganglia, internal capsule or thalamus may interfere with vestibulo-cortical signaling, leading to dizziness, which reflects the modulatory role of subcortical networks in spatial orientation.

The HINTS examination effectively distinguishes peripheral and central vertigo by assessing the head impulse test, nystagmus characteristics and ocular tilt reaction (22). This bedside tool is used in emergency neurology to rapidly evaluate AVS patients for potentially life-threatening central causes (particularly stroke) (22, 23). Some reports indicate HINTS demonstrates higher sensitivity than DWI for early stroke detection (24). Nevertheless, it may still miss over one-third of stroke-related dizziness cases (23, 25). In recent years, the development of quantitative vestibular function testing has provided an objective diagnostic basis for this challenging issue. Previous studies on the use of vestibular function tests to differentiate the etiology of acute vestibular syndrome have shown that the comprehensive application of vHIT and its saccade analysis, combined with tests such as videonystagmography (VNG), ocular vestibular evoked myogenic potential (oVEMP) and subjective visual horizontal (SVH), can enable early identification of posterior circulation stroke (26–28). In this study, most lesions involved only a single DWI slice with limited spatial extent, and half of patients had symptoms lasting merely hours. Notably, 61.5% (8/13) patients exhibited abnormal neuro-ophthalmological or neuro-otological findings, though manifestations were heterogeneous. 5 patients demonstrated abnormal eye movements, consistent with prior case studies (29). However, such central vestibular abnormalities do not occur in all supratentorial stroke patients, with nystagmus being an atypical sign in supratentorial strokes. While Park et al. reported normal vHITs and caloric testing in all 44 supratentorial stroke patients (7), four patients in our cohort showed abnormalities in these tests. Previous studies have found that disruptions in network connectivity predict various post-stroke dysfunctions, including executive and cognitive impairments (30–32). A possible explanation for this paradoxical peripheral involvement is that acute supratentorial stroke (particularly lesions involving the right parieto-insular vestibular cortex or the basal ganglia-thalamocortical circuit) induces remote dysfunction, leading to excitatory imbalance in the ipsilateral brainstem vestibular nuclei. This, in turn, produces clinical signs and symptoms resembling peripheral vestibular disorders. As the brain undergoes plastic repair, edema resolves, and contralateral brain regions compensate, the function of the vestibular nuclei gradually rebalances, and the clinical symptoms and abnormal signs subsequently improve or disappear. Unfortunately, this study did not include follow-up vestibular function tests. While these findings offer preliminary insight into the array of eye movement and vestibular function abnormalities that can be seen in supratentorial strokes, further research is needed to understand the potential utility of performing this additional testing to characterize supratentorial strokes presenting with AVS.

In summary, AVS induced by supratentorial stroke results from the interplay between multimodal network impairment and dynamic compensation (19, 21). For patients presenting with acute vertigo/dizziness, moving beyond the conventional HINTS protocol and incorporating neuro-ophthalmological/neuro-otological examinations is essential to enhance the accuracy of nystagmus assessment and reduce missed diagnoses of supratentorial stroke. This study has several limitations. First, as a single-center investigation with a small sample size, potential selection bias may limit generalizability. Therefore, future meta-analyses are needed to amalgamate the currently heterogeneous findings across the literature and overcome the limitations of individual studies with limited sample sizes. Second, ocular conditions or degenerative vestibular changes in elderly patients may lead to false-positive results. Reporting based on clinical routines may introduce reporting bias, and future studies should systematically collect and report all predefined evaluation indicators. Additionally, the disease onset timeline and medication effects on vestibular symptom resolution warrant further consideration. Finally, the mechanism underlying paradoxical peripheral vestibular involvement was not thoroughly explored, and future large-sample controlled studies incorporating DTI/fMRI to analyze vestibular connectivity could further elucidate the pathophysiological mechanisms of AVS in supratentorial stroke. Future research should consider a longitudinal follow-up design to compare vestibular function in the acute and recovery phases, which is essential for defining the evolution of observed abnormalities and understanding the disease’s long-term trajectory.

## Data Availability

The raw data supporting the conclusions of this article will be made available by the authors, without undue reservation.
